# Association of single nucleotide polymorphism at position −308 of the tumor necrosis factor-alpha gene with ankylosing spondylitis and rheumatoid arthritis

**DOI:** 10.1080/13102818.2014.972147

**Published:** 2014-11-07

**Authors:** Irena Manolova, Mariana Ivanova, Rumen Stoilov, Rasho Rashkov, Spaska Stanilova

**Affiliations:** ^a^ Department of Health Care, Faculty of Medicine, Trakia University, Stara Zagora, Bulgaria; ^b^ Clinic of Rheumatology, Department of Internal Medicine, University Hospital, Medical University of Sofia, Sofia, Bulgaria; ^c^ Department of Molecular Biology, Immunology and Medical Genetics, Faculty of Medicine, Trakia University, Stara Zagora, Bulgaria

**Keywords:** ankylosing spondylitis, promoter polymorphism, rheumatoid arthritis, cytokine

## Abstract

In this study, we analyzed the putative association between the −308 G/A polymorphism in the promoter region of the tumor necrosis factor (TNF) α gene (rs1800629) and chronic inflammatory arthritis in the Bulgarian population. A case-control study was carried out on 58 patients with ankylosing spondylitis (AS), 108 rheumatoid arthritis (RA) patients and 177 healthy subjects. −308 G/A TNF-α genotypes of patients and controls were determined by restriction fragment length polymorphism polymerase chain reaction (RFLP-PCR). No significant association between the rs1800629 polymorphism and RA risk in the study cohort was observed. However, there were significant differences in the genotype and allele frequencies of the −308 G/A TNF-α polymorphism between AS patients and the healthy subjects. In logistic regression analysis, the presence of the TNF-α −308A allele in the genotype (AA + AG vs. GG) was associated with a 3.298 times lower risk of developing AS. In addition, in AS, there were associations for age at disease onset (<29 years; odds ratio (OR) = 0.222), disease severity (Bath Ankylosing Spondylitis Disease Activity Index (BASDAI) score > 4; OR = 0.152) and response to anti-TNF treatment (OR = 2.25) under a dominant model (AA + AG vs. GG). In conclusion, our results suggested that the promoter polymorphism −308 G/A in the TNF-α gene had no significant effect on RA development, but could play a role in AS development and in determining the age of disease onset, disease severity and therapeutic outcome of AS in the Bulgarian patients who participated in our study.

## Introduction

Rheumatoid arthritis (RA) and ankylosing spondylitis (AS) are autoimmune rheumatic diseases in which chronic inflammation is associated with pathology of the peripheral joints or axial skeleton. RA is characterized by synovial joint inflammation and the overgrowth of synoviocytes, leading to cartilage and bone destruction. AS specifically involves the lower region of the spine and sacroiliac joints and causes enthesopathy and axial bone ankylosis. RA and AS are multifactorial diseases whose development depends on genetic as well as environmental factors. Genetic analysis of susceptibility to AS and RA suggests a polygenic inheritance pattern with the largest contribution from the major histocompatibility complex (MHC).[1,2] The MHC genetic contribution to these diseases is due to association of several alleles of the human leukocyte antigen (HLA)-DRB1 gene with RA and the HLA allele B27 with AS.

The prominent role played by tumor necrosis factor (TNF) α in inflammation and its relevance to AS and RA, as well as the location of the TNF-α gene (TNFA) within the class III region of MHC (between the HLA-B locus and HLA-DR) has led to great interest in the possibility that variants of the gene might be involved in disease susceptibility. Interindividual variations in the TNF production in healthy controls have been observed with high and low producer phenotypes, indicating a substantial genetic contribution to regulation of the TNF synthesis.[3,4] These findings suggest that polymorphism in the TNF-α regulatory region might influence its production. A number of single nucleotide polymorphisms (SNPs) within the promoter of the TNF-α gene has been identified. Among these common polymorphisms in the promoter, a G-to-A transition at position −308 (rs1800629) has been most widely analyzed.[5] Some investigations have suggested that such allelic variations could have functional significance, but the results obtained have been inconsistent.[6] Several studies have examined the potential contribution of −308 G/A TNFA SNP to AS and RA susceptibility.[7–14] However, the results about the relationship of the TNF-α rs1800629 polymorphism with RA and AS are still inconclusive.

The aim of this study was to determine whether the TNFA rs1800629 polymorphism contributes to the susceptibility to AS and RA and the characteristics of these diseases in the Bulgarian population. In addition, we also evaluated their possible use as predictors of the therapeutic response to TNF-α blockers.

## Subjects and methods

### Subjects

A total of 166 patients with AS and RA attending the Clinic of Rheumatology at St Ivan Rilski University Hospital (Sofia, Bulgaria) and 177 healthy subjects were included in this cross-sectional study. Of these patients, 58 had AS, according to the Modified New York criteria [15] and 108 had RA, according to the American College of Rheumatology criteria.[16] The longitudinal analysis included 17 patients with AS, in whom the improvement criteria for response to therapy at the sixth month were assessed (proportion of patients achieving 20% improvement by the Assessment of SpondyloArthritis International Society response criteria; ASAS20).

This study was approved by the institutional ethics committee and all subjects gave their informed consent.

### Clinical assessments

All participants completed extensive questionnaires about their medical history and rheumatologists performed a clinical evaluation on each participant. Clinical data included age, sex, disease duration and current medication history.

The Bath Ankylosing Spondylitis Disease Activity Index (BASDAI) [17] was used to measure patient-reported disease activity. BASDAI consists of six questions related to particular signs and symptoms of the disease (fatigue, spinal pain, pain in joints and entheses, morning stiffness severity and duration) and is completed by the patient on a 10-cm visual analogue scale (VAS). To determine the state of disease activity, we defined two levels for BASDAI. In our analysis, we used only the two most contrasting groups, where disease activity was defined as definite or no disease activity. A score under (<) 4 was assumed to mean no active disease, while a score above (≥) 4, that there was definite disease activity.

The outcome measure in AS was the proportion of patients showing a response based on ASAS20 at the sixth month of therapy. ASAS20 was calculated as a composite index: ≥20% relative improvement and absolute improvement of ≥1 units in at least three domains on a scale of 10, with no worsening in the fourth: inflammation (mean of BASDAI questions 5 and 6), function (Bath AS Functional Index), patient perception of pain on a 10-cm VAS and patient global assessment.[18]

Disease аctivity in RA was assessed by DAS28-ESR (Disease Activity Score 28-erythrocyte sedimentation rate). DAS28-ESR combines single measures into an overall, continuous measure of RA disease activity.[19–21] DAS28-ESR includes a 28 tender joint count, a 28 swollen joint count, ESR and a general health assessment on a 10-cm VAS.[19,21] Patients were subdivided into two groups on the basis of the DAS28-ESR score: lack of diseases activity (DAS28 ≤ 3.2) and presence of disease activity (DAS28 > 3.21).[22]

### Genotyping for −308 G/A polymorphism in TNFA

Genomic DNA was extracted from peripheral whole blood, using the NucleoSpin Blood L Purification kit (Macherey-Nagel, Duren, Germany) and stored at −80 °C until use. Genotyping for the −308G/A polymorphism in the TNFA (rs1800629) was performed by restriction fragment length polymorphism (RFLP) analysis of polymerase chain reaction (PCR) fragment amplified using the modified forward primer 5′-AGG CAA TAG GTT TTG AGG GCC AT-3′ and the reverse primer 5′-TTG GGG ACA CAC AAG CAT CAA GG-3′, to create a restriction site for the *NcoI* enzyme. PCR amplification was performed in a GeneAmp PCR System 9700 (Applied Biosystems). The thermocycling conditions were as follows: 95 °C for 2 min, 95 °C for 45 s, 65 °C for 45 s and 72 °C for 45 s, for 35 cycles and then 72 °C for 5 min. Overnight digestion with restriction enzyme *NcoI* (Thermo Scientific) was carried out with the PCR product at 37 °C and then analyzed in a 3% agarose gel. The sizes of PCR fragments were 150 bp for the −308 A allele and 128 bp and 22 bp for the −308 G allele.

### Statistical analysis

All data for this study were analyzed using the software SPSS version 16.0 for Windows (SPSS Inc., Chicago, IL). The differences in genotype distribution and allele frequency among cases and controls were analyzed using the χ^2^ test. The StatPages.org web site [23] was used to estimate odds ratios (ORs) expressed by their 95% confidence intervals (95% CI) for disease susceptibility and severity in relation to the −308 G/A TNFA polymorphism. The goodness of fit to the Hardy–Weinberg equilibrium, calculating the expected frequencies of each genotype and comparing them with the observed values for patients and healthy controls, was performed using a χ^2^ test. BASDAI and DAS28-ESR or subscores were used as continuous variables or as categorical variables upon categorization. Two-tailed *p*-values less than 0.05 were considered as significant.

## Results and discussion

### Characteristics of patients and healthy controls

A total of 58 patients with AS and 108 with RA were included in the study of distribution of the −308 G/A TNFA polymorphism. The demographic characteristics of the study participants, as well the clinical data of cases, are summarized in Table 1.

**Table 1.** Demographic and clinical data of study participants.

	AS (*n* = 58)	RA (*n* = 108)	Controls (*n* = 177)
Age (years)			
Mean (±SD)	38.1 ± 8.6	55.0 ± 11.2	47.5 ± 14
Range	22–57	22–78	19–83
Sex			
Male	46 (79.3%)	8 (7.4%)	85 (45%)
Female	12 (20.7%)	100 (92.6%)	92 (52%)
Disease duration (years)			
Mean (±SD)	10.9 ± 7.1	9.5 ± 7.9	
Range	1–30	1–40	
Drug treatment			
NSAIDs	19 (32.8%)	–	
sDMARDs	–	90 (83.4%)	
bDMARDs	27 (46.5%)	18 (16.6%)	
Disease activity	BASDAI score	DAS28-ESR	
Mean (±SD)	4.78 ± 2.24	5.04 ± 1.33	
Yes/no	34/24	69/39	

The group of the patients with AS consisted of 46 (79.3%) males and 12 (20.7%) females from 22 to 57 years of age with a mean (±SD, standard deviation) age of 38.1 ± 8.6 years. The mean (±SD) disease duration was 10.9 ± 7.1 years (range 1–30). Ninety-one per cent of AS patients were HLA-B27 positive. In terms of drug usage, 27 patients (46.5%) were treated with a TNF-α blocking agent (etanercept and adalimumab). Nineteen patients (32.8%) were taking a first-line therapy of non-steroidal anti-inflammatory drugs (NSAIDs). Thirty-four patients had active disease defined as BASDAI score ≥ 4.

The group of RA patients consisted of 8 (7.4%) males and 100 (92.6%) females from 22 to 78 years of age, with a mean (± SD) age of 55.0 ± 11.2 years. The mean (±SD) disease duration of RA was 9.5 ± 7.9 years (range 1–40). Eighteen patients (16.6%) were treated with biological disease modifying antirheumatic drugs (bDMARDs): two patients with TNF-blocking agents (etanercept and adalimumab) and 14 patients with IL-6RA (tocilizumab), at the time of the study; and the rest were taking synthetic DMARDs (sDMARDs) (methotrexate and sulfasalazine). In 69 RA patients with a DAS28-ESR score > 3.21, the disease was defined as active.

The sex and age distribution of the healthy controls was 85 (45%) males and 92 (52%) females; mean (±SD) age of 47.5 ± 14. years, range 19–83 years.

### Association of −308 G/A TNFA polymorphism with susceptibility to AS and RA

The genotype distribution and allele frequency of −308G/A in the gene promoter of TNFA among the cases and controls are presented in Table 2. The genotype distribution of the −308 G/A TNFA polymorphism among the AS cases was in agreement with the Hardy–Weinberg equilibrium (χ^2^ = 4.681; *p* = 0.09), RA cases (χ^2^ = 0.015; *p* = 0.992) and controls (χ^2^ = 0882; *p* = 0.643).

**Table 2.** Distribution of allele and genotype frequencies of the TNFA −308G/A polymorphism in the studied patients from Bulgaria.

rs1800629 TNF	AA	AG	AA/AG	GG		A	G
HC (*n* = 177)	2 (1.1%)	40 (22.6%)	42 (23.7%)	135 (76.3%)		44 (12.4%)	310 (87.6%)
AS (*n* = 58)	0 (0.0%)	5 (8.6%)	5 (8.6%)	53 (91.4%)	χ^2^ = 6.359 *p* = 0.042	5(4.3%)	111(95.7%)
OR (95% CI)		0.318 (0.104–0.903)	0.303 (0.099–0.89)	Reference		0.317 (0.108÷0.863)	Reference
*P*		0.029	0.021			0.021	
RA (*n* = 108)	1 (0.9%)	24 (22.2%)	25 (23.1%)	83 (76.9%)	χ^2^ = 0.34 *p* = 0.983	26 (12%)	190 (88%)
OR (95% CI)	0.813 (0.03–11.66)	0.976 (0.527–1.801)	0.968 (0.529–1.768)	Reference		0.964 (0.556/−1.665)	Reference
*P*	1.0	0.934	0.911			0.89	

Note: HC: healthy controls.

The genotype distribution of this polymorphism was almost identical between RA patients and controls (χ^2^ = 0.34; degrees of freedom, *df* = 2; *p* = 0.983). In the studied cohort, 76.9% of RA patients and 76.3% of control subjects were homozygous carriers of the GG genotype, heterozygous AG genotype was observed in 22.2% of RA patients and 22.6% of controls and homozygous AA genotype was observed in only 0.9% of RA cases and in 1.1% of controls. There were also no significant differences in the allele frequency of the −308 G/A TNFA polymorphism between RA patients and controls (χ^2^ = 0.015; *df* = 2; *p* = 0.89).

In contrast, we found significant differences in the genotype (χ^2^ = 6.359; *df* = 2; *p* = 0.042) and allele (χ^2^ = 5.238; *df* = 2; *p* = 0.021) frequencies of the −308 G/A TNFA polymorphism between AS patients and controls. There was higher frequency of the TNFA −308 G allele (95.7% vs. 87.6%; OR = 3.151) and lower frequency of the TNFA −308 A allele in AS patients vs. healthy controls (4.3% vs. 12.4%; OR = 0.317). A higher frequency of the GG genotype (91.4%) and lower frequency of heterozygous AG genotype (8.6%) was found in AS patients as compared to the controls. Logistic regression analysis revealed that the presence of the TNFA −308 A allele in the genotype (AA + AG vs. GG) was associated with a 3.298 times lower risk of developing AS (OR = 0.303; 95% CI 0.099–0.58; *p* = 0.021). These results suggest that the presence of the TNFA −308 minor allele A in the genotype could be protective to susceptibility of AS.

### Role of the −308 G/A TNFA polymorphism in clinical manifestation of AS and RA

The genotype distribution of SNP in the −308 locus of TNFA among the studied patients was examined in relation to some clinical parameters. An association was found in AS for age at disease onset (age at onset < 29 years; OR = 0.222; 95% CI 0.04–1.02; *p* = 0.05) as well as for disease severity (BASDAI ≥ 4; OR = 0.152, 95% CI 0.006–1.633, *p* = 0.067) under a dominant model (AA + AG vs. GG) (Table 3). These results suggest that carriage of the TNFA −308 A allele in the genotype is a protective factor for early appearance of AS (below 29 years of age) and manifestation of most severe disease (BASDAI ≥ 4).

**Table 3.** Clinical data of AS patients in correlation with the TNFA −308G/A polymorphism genotypes.

	GG, *n* (%)	AA + AG, *n* (%)
	Age at onset	
<29 years	29 (54.7)	2 (40.0)
>29 years	24 (45.3)	3 (60.0)
	OR = 0.222; 95% CI 0.04–1.02	
	Disease severity	
BASDAI < 4	20 (37.7)	4 (80.0)
BASDAI ≥ 4	33 (62.3)	1 (20.0)
	OR = 0.152; 95% CI 0.006–1.633	
	Response to anti-TNF treatment (ASAS20)	
No	9 (69.3)	2 (50)
Yes	4 (30.7)	2 (50)
	OR = 2.25; 95% CI 0.144–38.542	

Note: Only odds ratios (ORs) for AA + AG are shown.

We also found that the presence or absence of an A allele at locus −308 was related to the clinical outcome of anti-TNF-α treatment in AS patients. Fifty per cent of the patients carrying the −308A allele in the genotype achieved improvement, according to the ASAS20 under the criteria assessing the response to therapy, whereas for the patients carrying the −308 GG genotype, there was improvement only in 30.7% (Table 3). As the number of TNFA −308 AG heterozygotes was small, the difference between −308 AG heterozygotes and −308 GG homozygotes in regard to their response to anti-TNA-α treatment was not statistically significant.

No association of the −308 G/A TNFA polymorphism with age of onset, disease severity (assessed by the DAS28 score) and response to therapy in RA was found (data not shown).

Because the TNF-α gene encodes one of the most potent proinflammatory cytokine and immune modulator of joint destruction and due to the localization of the TNF-α gene within the MHC in close vicinity of the HLA-B locus, the TNF-α gene constitutes an important candidate for determining genetic susceptibility to inflammatory rheumatic disease, such as RA and AS. Also, the first TNF-α promoter SNP identified (the −308 G-to-A transition) is strongly associated with the HLA-A1-B8-DR3 haplotype,[24] known to predispose to various autoimmune diseases.

The TNFA −308 G/A polymorphism has been reported to be associated with AS, but the results reported are contradictory.[25] Two studies performed in AS patients from Germany [10] and Scotland [12] established reduced incidence of the −308 A allele in AS and have concluded that allelic variants in the TNFA promoter influence disease susceptibility in HLA-B27 positive individuals. In the southern German AS patients, a significant reduction in the TNFA −308 A allele was also reported, but no difference in allele frequencies was observed in the English population.[13] As well, two other studies involving Spanish AS patients failed to identify significant differences of the TNFA promoter variations at position −308 between the AS cases and controls.[9,26]

In this study, we found under-representation of the TNFA −308 A allele in Bulgarian patients with AS. Our results suggest that the presence of the TNFA −308 minor allele A in the genotype could be protective against susceptibility to AS. In addition, the carriage of the TNFA −308 A allele could be a protective factor against early appearance of AS and manifestation of most severe disease, thus having a modifying effect on the clinical presentation of the disease.

Our study, however, has some limitations: first, the HLA-B27 status of the healthy controls subjected to analysis was not known, whereas 91% of the studied AS patients were HLA-B27 positive; second, the number of −308 G/A TNFA heterozygotes was relatively small. Further studies are needed to explain the molecular mechanism underlying the significance of the A allele for protection against AS development and severity.

Although the therapeutic options for patients with active and severe AS are still fairly limited, there is accumulating evidence that biologic therapy with agents directed against TNF-α is highly efficacious.[27] It has been suggested that TNF-α overexpression acts as a driver for inflammation that damages cartilage and bone, and TNF-α inhibition leads to significant clinical improvement and reduction of this damage.[28] Studies in patients with RA, psoriatic arthritis or AS suggested that patients with TNFA −308 GG genotype are better responders to anti-TNF-α treatment than those with AA or AG genotypes.[29] In our study, we found that the proportion of patients that showed a therapeutic response was higher among the −308 A/G heterozygotes as compared to the GG homozygotes. However, these results should be interpreted with caution due to the lower statistical power of the observation and the lack of statistically significant difference in the allelic frequency between responders and non-responders.

Several studies have examined the association of the TNFA SNP at locus −308 and the susceptibility to RA. Wilson et al. [30] for the first time examined the −308 G/A SNP in relation to RA and they did not establish an association of TNFA alleles with susceptibility to RA for the Dutch population. Also, in Spanish patients, two studies found no association of the TNFA promoter variation at position −308 with RA susceptibility.[11,14] However, unlike the Caucasians, in a large cohort of Japanese patients with RA (*n* = 545), an increased frequency of the −308 G allele was established, which was associated with HLA-DRB1*0405.[31] Another study with a non-Caucasian population from Taiwan [32] also showed an increased frequency of the −308 G allele in RA patients. It could be considered that the −308 G/A SNP may have a different effect on the genetic predisposition to RA development, depending on the race. Our results are in agreement with these observations, as Bulgarians belong to Caucasians.

The influence of allelic variants at locus −308 of TNFA on the development and severity of RA has also been evaluated in addition to studies on their frequencies.[7,33] In Swedish patients, it has been demonstrated that individuals bearing the heterozygous TNFA −308 AG genotype develop a more severe disease with an earlier onset,[7] while in Turkish RA patients, a significant association with bad prognosis of the disease has also been described.[33] As summarized by Lee and Song [25], a number of factors, such as clinical heterogeneity, ethnic differences or real genetic heterogeneity, small sample size of the studies or their low statistical power may explain the controversial data in the literature. Our data showed no association of the studied genotypes (the TNF-α −308 A/G polymorphisms) and RA severity. The lack of influence of −308 TNF-α gene polymorphism on the severity of RA has already been reported by others.[5,34] Subsequently, the analysis performed after stratification by age at onset detected no significant association with the TNFA −308A/G polymorphism.

## Conclusions

The results from this study showed that the TNFA rs1800629 gene polymorphism is associated with the genetic susceptibility to ankylosing spondylitis, age at onset and disease severity in the Bulgarian population. In contrast to AS, an apparent association was not shown between the −308 A/G polymorphism of the TNF-α gene and susceptibility to RA in the studied population. These data support the hypothesis for the different pathogenetic mechanisms underlying both these inflammatory joint diseases. In addition, the TNFA promoter polymorphism at locus −308 could affect the therapeutic outcome in response to biological agents, such as monoclonal antibodies against cytokines and cytokine receptors, in AS patients.

## References

[cit0001] Brown MA, Rudwaleit M, Pile KD, Kennedy LG, Shatford J, Amos CI, Siminovitch K, Rubin L, Calin A, Wordsworth BP (1998). The role of germline polymorphisms in the T-cell receptor in susceptibility to ankylosing spondylitis. Br J Rheumatol.

[cit0002] Cornélis F, Fauré S, Martinez M, Prud'homme JF, Fritz P, Dib C, Alves H, Barrera P, de Vries N, Balsa A, Pascual-Salcedo D, Maenaut K, Westhovens R, Migliorini P, Tran TH, Delaye A, Prince N, Lefevre C, Thomas G, Poirier M, Soubigou S, Alibert O, Lasbleiz S, Fouix S, Bouchier C, Lioté F, Loste MN, Lepage V, Charron D, Gyapay G, Lopes-Vaz A, Kuntz D, Bardin T, Weissenbach J (1998). New susceptibility locus for rheumatoid arthritis suggested by a genome-wide linkage study. Proc Natl Acad Sci USA.

[cit0003] Louis E, Franchimont D, Piron A, Gevaert Y, Schaaf-Lafontaine N, Roland S, Mahieu P, Malaise M, De Groote D, Louis R, Belaiche J (1998). Tumour necrosis factor (TNF) gene polymorphism influences TNF-alpha production in lipopolysaccharide (LPS)-stimulated whole blood cell culture in healthy humans. Clin Exp Immunol.

[cit0004] Bayley JP, Ottenhoff TH, Verweij CL (2004). Is there a future for TNF promoter polymorphisms. Genes Immun.

[cit0005] Mugnier B, Balandraud N, Darque A, Roudier C, Roudier J, Reviron D (2003). Polymorphism at position -308 of the tumor necrosis factor alpha gene influences outcome of infliximab therapy in rheumatoid arthritis. Arthritis Rheum.

[cit0006] Bouma G, Crusius JB, Oudkerk Pool M, Kolkman JJ, von Blomberg BM, Kostense PJ, Giphart MJ, Schreuder GM, Meuwissen SG, Peña AS (1996). Secretion of tumour necrosis factor alpha and lymphotoxin alpha in relation to polymorphisms in the TNF genes and HLA-DR alleles. Relevance for inflammatory bowel disease. Scand J Immunol.

[cit0007] Cvetkovic JT, Wallberg-Jonsson S, Stegmayr B, Rantapaa-Dahlqvist S, Lefvert AK (2002). Susceptibility for and clinical manifestations of rheumatoid arthritis are associated with polymorphisms of the TNF-alpha, IL-1beta, and IL-1Ra genes. J Rheumatol.

[cit0008] Danis VA, Millington M, Hyland V, Lawford R, Huang Q, Grennan D (1995). Increased frequency of the uncommon allele of a tumour necrosis factor alpha gene polymorphism in rheumatoid arthritis and systemic lupus erythematosus. Dis Markers.

[cit0009] Fraile A, Nieto A, Beraún Y, Vinasco J, Matarán L, Martín J (1998). Tumor necrosis factor gene polymorphisms in ankylosing spondylitis. Tissue Antigens.

[cit0010] Höhler T, Schäper T, Schneider PM, Meyer zum Büschenfelde KH, Märker-Hermann E (1998). Association of different tumor necrosis factor alpha promoter allele frequencies with ankylosing spondylitis in HLA-B27 positive individuals. Arthritis Rheum.

[cit0011] Martínez A, Fernández-Arquero M, Pascual-Salcedo D, Conejero L, Alves H, Balsa A, de la Concha EG (2000). Primary association of tumor necrosis factor-region genetic markers with susceptibility to rheumatoid arthritis. Arthritis Rheum.

[cit0012] McGarry F, Walker R, Sturrock R, Field M (1999). The -308.1 polymorphism in the promoter region of the tumor necrosis factor gene is associated with ankylosing spondylitis independent of HLA-B27. J Rheumatol.

[cit0013] Milicic A, Lindheimer F, Laval S, Rudwaleit M, Ackerman H, Wordsworth P, Hohler T, Brown MA (2000). Interethnic studies of TNF polymorphisms confirm the likely presence of a second MHC susceptibility locus in ankylosing spondylitis. Genes Immun.

[cit0014] Vinasco J, Beraún Y, Nieto A, Fraile A, Mataran L, Pareja E, Martín J (1997). Polymorphism at the TNF loci in rheumatoid arthritis. Tissue Antigens.

[cit0015] van der Linden S, Valkenburg HA, Cats A (1984). Evaluation of diagnostic criteria for ankylosing spondylitis. A proposal for modification of the New York criteria. Arthritis Rheum.

[cit0016] Arnett FC, Edworthy SM, Bloch DA, McShane DJ, Fries JF, Cooper NS, Healey LA, Kaplan SR, Liang MH, Luthra HS, Medsger TA, Mitchell DM, Neustadt DH, Pinals RS, Schaller JG, Sharp JT, Wilder RL, Hunder GG (1988). The American Rheumatism Association 1987 revised criteria for the classification of rheumatoid arthritis. Arthritis Rheum..

[cit0017] Garrett S, Jenkinson T, Kennedy LG, Whitelock H, Gaisford P, Calin A (1994). A new approach to defining disease status in ankylosing spondylitis: the Bath Ankylosing Spondylitis Disease Activity Index. J Rheumatol.

[cit0018] Anderson JJ, Baron G, van der Heijde D, Felson DT, Dougados M (2001). Ankylosing spondylitis assessment group preliminary definition of short-term improvement in ankylosing spondylitis. Arthritis Rheum.

[cit0019] Prevoo ML, van ‘t Hof MA, Kuper HH, van Leeuwen MA, van de Putte LB, van Riel PL (1995). Modified disease activity scores that include twenty-eight-joint counts. Development and validation in a prospective longitudinal study of patients with rheumatoid arthritis. Arthritis Rheum.

[cit0020] van der Heijde DM, van ‘t Hof MA, van Riel PL, Theunisse LA, Lubberts EW, van Leeuwen MA, van Rijswijk MH, van de Putte LB (1990). Judging disease activity in clinical practice in rheumatoid arthritis: first step in the development of a disease activity score. Ann Rheum Dis.

[cit0021] van Riel PL, Schumacher HR Jr (2001). How does one assess early rheumatoid arthritis in daily clinical practice. Best Pract Res Clin Rheumatol.

[cit0022] van Gestel AM, Haagsma CJ, van Riel PL (1998). Validation of rheumatoid arthritis improvement criteria that include simplified joint counts. Arthritis Rheum.

[cit0023] The StatPages.org Internet.

[cit0024] Wilson AG, de Vries N, Pociot F, Giovine FS, van der Putte LB, Duff GW (1993). An allelic polymorphism within the human tumor necrosis factor alpha promoter region is strongly associated with HLA A1, B8, and DR3 alleles. J Exp Med.

[cit0025] Lee YH, Song GG (2009). Lack of association of TNF-alpha promoter polymorphisms with ankylosing spondylitis: a meta-analysis. Rheumatology (Oxford).

[cit0026] Martinez-Borra J, Gonzalez S, López-Vazquez A, Gelaz MA, Armas JB, Kanga U, Mehra NK, López-Larrea C (2000). HLA-B27 alone rather than B27-related class I haplotypes contributes to ankylosing spondylitis susceptibility. Hum Immunol.

[cit0027] Braun J, Davis J, Dougados M, Sieper J, van der Linden S, van der Heijde D, ASAS Working Group (2006). First update of the international ASAS consensus statement for the use of anti-TNF agents in patients with ankylosing spondylitis. Ann Rheum Dis.

[cit0028] Nash PT, Florin TH (2005). Tumour necrosis factor inhibitors. Med J Aust..

[cit0029] Seitz M, Wirthmüller U, Möller B, Villiger PM (2007). The -308 tumour necrosis factor-alpha gene polymorphism predicts therapeutic response to TNFalpha-blockers in rheumatoid arthritis and spondyloarthritis patients. Rheumatology (Oxford).

[cit0030] Wilson AG, de Vries N, van de Putte LB, Duff GW (1995). A tumour necrosis factor alpha polymorphism is not associated with rheumatoid arthritis. Ann Rheum Dis.

[cit0031] Shibue T, Tsuchiya N, Komata T, Matsushita M, Shiota M, Ohashi J, Wakui M, Matsuta K, Tokunaga K (2000). Tumor necrosis factor alpha 5’-flanking region, tumor necrosis factor receptor II, and HLA-DRB1 polymorphisms in Japanese patients with rheumatoid arthritis. Arthritis Rheum.

[cit0032] Yen JH, Chen CJ, Tsai WC, Lin CH, Ou TT, Wu CC, Liu HW (2001). Tumor necrosis factor promoter polymorphisms in patients with rheumatoid arthritis in Taiwan. J Rheumatol.

[cit0033] Ozen S, Alikasifoglu M, Bakkaloglu A, Duzova A, Jarosova K, Nemcova D, Besbas N, Vencovsky J, Tuncbilek E (2002). Tumour necrosis factor alpha G–>A -238 and G–>A -308 polymorphisms in juvenile idiopathic arthritis. Rheumatology (Oxford).

[cit0034] Lacki JK, Moser R, Korczowska I, Mackiewicz S, Muller W (2000). TNF-alpha gene polymorphism does not affect the clinical and radiological outcome of rheumatoid arthritis. Rheumatol Int.

